# Supplementary material for: DOT1L provides transcriptional memory through PRC1.1 antagonism
Source: Nat Cell Biol. 2026 Feb 3;28(2):307–22. doi: 10.1038/s41556-025-01859-8 (PMC12904788; doi:10.1038/s41556-025-01859-8)
Supplement: Supplementary file 1 — Reporting Summary [file 41556_2025_1859_MOESM1_ESM.pdf]

nature portfolio

Corresponding author(s): Omer Gilan

Last updated by author(s): Nov 24, 2025

Reporting Summary

Nature Portfolio wishes to improve the reproducibility of the work that we publish. This form provides structure for consistency and transparency in reporting. For further information on Nature Portfolio policies, see our Editorial Policies and the Editorial Policy Checklist.

Statistics

For all statistical analyses, confirm that the following items are present in the figure legend, table legend, main text, or Methods section.

n/a	Confirmed	
☐	☒	The exact sample size (*n*) for each experimental group/condition, given as a discrete number and unit of measurement
☐	☒	A statement on whether measurements were taken from distinct samples or whether the same sample was measured repeatedly
☐	☒	The statistical test(s) used AND whether they are one- or two-sided<br>*Only common tests should be described solely by name; describe more complex techniques in the Methods section.*
☒	☐	A description of all covariates tested
☐	☒	A description of any assumptions or corrections, such as tests of normality and adjustment for multiple comparisons
☐	☒	A full description of the statistical parameters including central tendency (e.g. means) or other basic estimates (e.g. regression coefficient) AND variation (e.g. standard deviation) or associated estimates of uncertainty (e.g. confidence intervals)
☐	☒	For null hypothesis testing, the test statistic (e.g. *F*, *t*, *r*) with confidence intervals, effect sizes, degrees of freedom and *P* value noted<br>*Give P values as exact values whenever suitable.*
☒	☐	For Bayesian analysis, information on the choice of priors and Markov chain Monte Carlo settings
☒	☐	For hierarchical and complex designs, identification of the appropriate level for tests and full reporting of outcomes
☐	☒	Estimates of effect sizes (e.g. Cohen's *d*, Pearson's *r*), indicating how they were calculated

Our web collection on statistics for biologists contains articles on many of the points above.

Software and code

Policy information about availability of computer code

Data collection

Flow cytometry data was collected on either on BD LSRII or LSRFortessa (BD Biosciences), using DiVA V6.3.1.
Sequencing data was collected on either the NextSeq550 or NovaSeq6000 (Illumina) using Basespace to convert BCL files to FastQs

Data analysis

All murine sequencing data were aligned to mm10 and human sequencing data were aligned to hg38

CRISPR Screen
CRISPR screen data was analysed using MAGeCK ver. 0.5.9.4 and then plotted using Prism ver. 9

ChIP-seq
Reads were mapped using Bowtie2, BAM files were converted to TDFs using igvtools ver. 2.4.19 and bigwigs (BamCoverage), then heatmap and profile plots were made using deepTools ver. 3.1.3. Peaks were called using MACS2 ver. 2.1.1.2016309.

RNA-seq
Reads were trimmed using bbmap ver. 38.81 and aligned using STAR aligner ver. 2.7.9a and then fed into Degust for differential expression analysis.

RStudio
All RNA-seq and the non-deepTools ChIP-seq plots were made using RStudio ver. 4.0.2 with the following packages: biomaRt ver. 2.54.1, BioVenn ver. 1.1.3, BSgenome.Mmusculus.UCSC.mm10 ver. 1.4.3, ChIPpeakAnno ver. 3.32.0, csaw ver. 1.32.0, dplyr ver. 1.1.3, ggplot2 ver. 3.4.4, ggpubr ver. 0.6.0, ggrepel ver. 0.9.4, org.Hs.eg.db ver. 3.16.0, org.Mm.eg.db ver. 3.16.0, pheatmap ver. 1.0.12, profileplyR ver.. 1.14.1, reshape2 ver. 1.4.4, TxDb.Hsapiens.UCSC.hg38.knownGene ver 3.16.0, TxDb.Mmusculus.UCSC.mm10.ensGene ver. 3.4.0

Flow Cytometry
FlowLogic ver. 1.3 and FlowJo ver. 10

For manuscripts utilizing custom algorithms or software that are central to the research but not yet described in published literature, software must be made available to editors and reviewers. We strongly encourage code deposition in a community repository (e.g. GitHub). See the Nature Portfolio guidelines for submitting code & software for further information.

Data

Policy information about availability of data

All manuscripts must include a data availability statement. This statement should provide the following information, where applicable:

- Accession codes, unique identifiers, or web links for publicly available datasets
- A description of any restrictions on data availability
- For clinical datasets or third party data, please ensure that the statement adheres to our policy

MLL1 ChIP-seq data in K562 cells (Extended Data Fig. 8b) GEO Accession Code GSE181829
RNA-seq data produced for this manuscript GEO Accession Code GSE260456
ChIP-seq data produced for this manuscript GEO Accession Code GSE260742

Research involving human participants, their data, or biological material

Policy information about studies with human participants or human data. See also policy information about sex, gender (identity/presentation), and sexual orientation and race, ethnicity and racism.

Reporting on sex and gender	N/A
Reporting on race, ethnicity, or other socially relevant groupings	N/A
Population characteristics	N/A
Recruitment	N/A
Ethics oversight	N/A

Note that full information on the approval of the study protocol must also be provided in the manuscript.

Field-specific reporting

Please select the one below that is the best fit for your research. If you are not sure, read the appropriate sections before making your selection.

☒ Life sciences ☐ Behavioural & social sciences ☐ Ecological, evolutionary & environmental sciences

For a reference copy of the document with all sections, see nature.com/documents/nr-reporting-summary-flat.pdf

Life sciences study design

All studies must disclose on these points even when the disclosure is negative.

Sample size	No statistical method was used to determine sample sizes and was selected prior to knowledge of the outcome. No power analysis was carried out.
Data exclusions	No data were excluded from this study except for those that were clear outliers due to technical problems in the assays.
Replication	Replicates are indicated in the figure legends and/or methods. Most assays were performed in at least biological triplicate. All experiments were able to be reliably reproduced.
Randomization	Mice were randomly assigned to cohorts at beginning of treatment. Cells were randomly split from the same pool of cells before being subject to treatment. Randomisation was applicable to other experiments.
Blinding	In vivo experiments utilized blinded technicians for assessing disease severity at end point. Investigators were not blinded to allocation during experiments and outcome assessment.

Reporting for specific materials, systems and methods

We require information from authors about some types of materials, experimental systems and methods used in many studies. Here, indicate whether each material, system or method listed is relevant to your study. If you are not sure if a list item applies to your research, read the appropriate section before selecting a response.

Materials & experimental systems

n/a	Involved in the study
☐	☒ Antibodies
☐	☒ Eukaryotic cell lines
☒	☐ Palaeontology and archaeology
☐	☒ Animals and other organisms
☒	☐ Clinical data
☒	☐ Dual use research of concern
☒	☐ Plants

Methods

n/a	Involved in the study
☐	☒ ChIP-seq
☐	☒ Flow cytometry
☒	☐ MRI-based neuroimaging

Antibodies

Antibodies used

DYKDDDDK Tag (D6W5B) Rabbit mAb (New England Biolabs 14793S)
Rabbit anti-MLL1 Antibody (Bio-Strategy BETHA300-086A-M)
Anti-trimethyl-Histone H3 (Lys4) (Merck 07-473)
Rabbit polyclonal to Histone H3 (di methyl K79) - ChIP Grade (ABCAM ab3594-100UG)
Rabbit polyclonal to Histone H3 (acetyl K9) (ABCAM ab4441-100UG)
Rabbit polyclonal to Histone H3 (acetyl K27) (ABCAM ab4729-100UG)
Ubiquityl-Histone H2B (Lys120) (D11) XP® Rabbit mAb (New England Biolabs 5546S)
RING1B (D22F2) XP® Rabbit mAb (New England Biolabs 5694S)
ChIPAb+™ JHDM1B Antibody (Merck 17-10264)
Ubiquityl-Histone H2A (Lys119) (D27C4) XP® Rabbit mAb (New England Biolabs 8240S)
Tri-Methyl-Histone H3 (Lys27) (C36B11) Rabbit mAb (New England Biolabs 9733S)
α-Tubulin (11H10) Rabbit mAb (HRP Conjugate) (New England Biolabs 9099S)
anti-mouse/human CD11b, APC-conjugated (BioLegend 301310)
Anti-human CD45, APC/Cy7-conjugated (BioLegend 304014)
Anti-mouse Ly6G Ly6C, FITC (Becton Dickinson 562060)
Anti-human CD11b, APC/Cy7 (Becton Dickinson 56103)
FITC Mouse Anti-Human CD235a BD Pharminigen 559943
PE/Cyanine7 Anti-Human CD49d BioLegend 304313
BCOR Rabbit Polyclonal antibody United BioResearch 12107-1-AP
Rabbit polyclonal Histone H3 ABCAM ab1791
H2AK119ub (for ubiquitination assay) CST D27C4
HRP-conjugated goat anti-rabbit Abcam A0545
Rabbit anti-H3 Abcam Ab1791
Mouse anti-MBP NEB E8032L
HRP-conjugated donkey anti-mouse Jackson Immuno Research 715-035-150

Validation

All antibodies used have been validated in previous publications according to manufacturer's websites.

Eukaryotic cell lines

Policy information about cell lines and Sex and Gender in Research

Cell line source(s)

MLLAF9 leukaemia cells were generated by magnetic bead selection (Miltenyi Biotec) of c-KIT+ cells from whole female mouse bone marrow and retroviral transduction with MLLAF9 constructs. MOLM13, MV4;11, K562, OCI-AML3 and HEK293T cells were obtained from ATCC or DSMZ. Murine embryonic stem cells and HuDEP-2 cells were obtained from collaborators.

Authentication

STR testing was performed to authenticate commercial cell lines

Mycoplasma contamination

Cell lines routinely tested negative for mycoplasma contamination.

Commonly misidentified lines
(See ICLAC register)

No commonly misidentified cell lines were used in the study.

Animals and other research organisms

Policy information about studies involving animals; ARRIVE guidelines recommended for reporting animal research, and Sex and Gender in Research

Laboratory animals

Female NOG Mice (NOD.Cg-Prkdcscidll2rgtm1Wjl/SzJ stock#005557) mice at 6-8 weeks of age

Wild animals

No wild animals were used.

Reporting on sex	To minimise variation in each mouse experiment, animals were all females.
Field-collected samples	No field samples were collected.
Ethics oversight	Animal experiments were approved by the Dana-Farber Cancer Institute's Institutional Animal Care and Use Committee.

Note that full information on the approval of the study protocol must also be provided in the manuscript.

Plants

Seed stocks	N/A
Novel plant genotypes	N/A
Authentication	N/A

ChIP-seq

Data deposition

☒ Confirm that both raw and final processed data have been deposited in a public database such as GEO.

☒ Confirm that you have deposited or provided access to graph files (e.g. BED files) for the called peaks.

Data access links
May remain private before publication.

To review GEO accession GSE260742

Files in database submission

GSM8123342 HUDEP-2 Control - DMSO input
GSM8123343 HUDEP-2 Control - DMSO H2KAK119ub
GSM8123344 HUDEP-2 Control - DMSO H3K79me2
GSM8123345 HUDEP-2 Control - SGC0946 5uM H2AK119ub
GSM8123346 HUDEP-2 Control - SGC0946 5uM H3K79me2
GSM8123347 K562 Control - DMSO input
GSM8123348 K562 Control - DMSO H2KAK119ub
GSM8123349 K562 Control - DMSO H3K79me2
GSM8123350 K562 Control - SGC0946 5uM H2AK119ub
GSM8123351 K562 Control - SGC0946 5uM H3K79me2
GSM8123352 K562 Control - VTP50469 500nM H2AK119ub
GSM8123353 K562 Control - VTP50469 500nM H3K79me2
GSM8123354 ESC Control - DMSO input
GSM8123355 ESC Control - DMSO H2KAK119ub
GSM8123356 ESC Control - DMSO H3K79me2
GSM8123357 ESC Control - SGC0946 5uM H2AK119ub
GSM8123358 ESC Control - SGC0946 5uM H3K79me2
GSM8123359 MLLAF9 Control - DMSO FLAG
GSM8123360 MLLAF9 Control - SGC0946 5uM FLAG
GSM8123361 MLLAF9 Control - VTP50469 500nM FLAG
GSM8123362 MLLAF9 Control - DMSO input
GSM8123363 MLLAF9 Control - DMSO input2
GSM8123364 MLLAF9 Control - SGC0946 5uM VTP50469 500nM H2AK119ub
GSM8123365 MLLAF9 Control - DMSO H2AK119ub
GSM8123366 MLLAF9 Control - SGC0946 5uM H2AK119ub
GSM8123367 MLLAF9 Control - VTP50469 500nM H2AK119ub
GSM8123368 MLLAF9 Control - SGC0946 5uM VTP50469 500nM H3K27me3
GSM8123369 MLLAF9 Control - DMSO H3K27me3
GSM8123370 MLLAF9 Control - SGC0946 5uM H3K27me3
GSM8123371 MLLAF9 Control - VTP50469 500nM H3K27me3
GSM8123372 MLLAF9 Control - DMSO H3K79me2
GSM8123373 MLLAF9 Control - SGC0946 5uM H3K79me2
GSM8123374 MLLAF9 Control - VTP50469 500nM H3K79me2
GSM8123375 MLLAF9 Control - DMSO input for ac
GSM8123376 MLLAF9 Control - DMSO input for RING1B
GSM8123377 MLLAF9 Control - DMSO H2AK119ub rep2
GSM8123378 MLLAF9 Control - SGC0946 5uM H2AK119ub rep2
GSM8123379 MLLAF9 Control - VTP50469 500nM H2AK119ub rep2
GSM8123380 MLLAF9 Control - DMSO H2AK120ub

GSM8123381 MLLAF9 Control - SGC0946 5uM H2AK120ub
GSM8123382 MLLAF9 Control - VTP50469 500nM H2AK120ub
GSM8123383 MLLAF9 Control - DMSO H3K27ac
GSM8123384 MLLAF9 Control - SGC0946 5uM H3K27ac
GSM8123385 MLLAF9 Control - VTP50469 500nM H3K27ac
GSM8123386 MLLAF9 Control - DMSO H3K79me2 rep2
GSM8123387 MLLAF9 Control - SGC0946 5uM H3K79me2 rep2
GSM8123388 MLLAF9 Control - VTP50469 500nM H3K79me2 rep2
GSM8123389 MLLAF9 Control - DMSO H3K9ac
GSM8123390 MLLAF9 Control - SGC0946 5uM H3K9ac
GSM8123391 MLLAF9 Control - VTP50469 500nM H3K9ac
GSM8123392 MLLAF9 Control - DMSO KDM2B
GSM8123393 MLLAF9 Control - SGC0946 5uM KDM2B
GSM8123394 MLLAF9 Control - VTP50469 500nM KDM2B
GSM8123395 MLLAF9 Control - DMSO MLL1
GSM8123396 MLLAF9 Control - SGC0946 5uM MLL1
GSM8123397 MLLAF9 Control - VTP50469 500nM MLL1
GSM8123398 MLLAF9 Control - DMSO RING1B
GSM8123399 NTvKO PCGF1 KO DMSO H2AK119ub paired
GSM8123400 NTvKO PCGF1 KO DMSO input paired
GSM8123401 NTvKO PCGF1 KO SGC0946 5uM H2AK119ub paired
GSM8123402 NTvKO PCGF1 KO VTP50469 500nM H2AK119ub paired
GSM8123403 NTvKO Control - DMSO H2AK119ub paired
GSM8123404 NTvKO Control - DMSO input paired
GSM8123405 NTvKO Control - SGC0946 5uM H2AK119ub paired
GSM8123406 NTvKO Control - VTP50469 500nM H2AK119ub paired
GSM8123407 NTvKO PCGF1 KO DMSO H2AK119ub rep1
GSM8123408 NTvKO PCGF1 KO SGC0946 5uM H2AK119ub rep1
GSM8123409 NTvKO PCGF1 KO VTP50469 500nM H2AK119ub rep1
GSM8123410 NTvKO Control - DMSO H2AK119ub rep1
GSM8123411 NTvKO Control - SGC0946 5uM H2AK119ub rep1
GSM8123412 NTvKO Control - VTP50469 500nM H2AK119ub rep1
GSM8123413 NTvKO Control - DMSO input1
GSM8123414 NTvKO Control - DMSO input2
GSM8123415 NTvKO PCGF1 KO DMSO input
GSM8123416 NTvKO Control - DMSO input3
GSM8123417 NTvKO PCGF1 KO DMSO H2AK119ub rep2
GSM8123418 NTvKO PCGF1 KO SGC0946 5uM H2AK119ub rep2
GSM8123419 NTvKO PCGF1 KO VTP50469 500nM H2AK119ub rep2
GSM8123420 NTvKO Control - DMSO H2AK119ub rep2
GSM8123421 NTvKO Control - SGC0946 5uM H2AK119ub rep2
GSM8123422 NTvKO Control - VTP50469 500nM H2AK119ub rep2
GSM8123423 NTvKO PCGF1 KO DMSO H3K27me3
GSM8123424 NTvKO PCGF1 KO SGC0946 5uM H3K27me3
GSM8123425 NTvKO PCGF1 KO VTP50469 500nM H3K27me3
GSM8123426 NTvKO Control - DMSO H3K27me3
GSM8123427 NTvKO Control - SGC0946 5uM H3K27me3
GSM8123428 NTvKO Control - VTP50469 500nM H3K27me3
GSM8123429 NTvKO PCGF1 KO DMSO H3K4me3
GSM8123430 NTvKO PCGF1 KO SGC0946 5uM H3K4me3
GSM8123431 NTvKO PCGF1 KO VTP50469 500nM H3K4me3
GSM8123432 NTvKO Control - DMSO H3K4me3
GSM8123433 NTvKO Control - SGC0946 5uM H3K4me3
GSM8123434 NTvKO Control - VTP50469 500nM H3K4me3
GSM8123435 NTvKO PCGF1 KO DMSO H3K79me2
GSM8123436 NTvKO PCGF1 KO SGC0946 5uM H3K79me2
GSM8123437 NTvKO PCGF1 KO VTP50469 500nM H3K79me2
GSM8123438 NTvKO Control - DMSO H3K79me2
GSM8123439 NTvKO Control - SGC0946 5uM H3K79me2
GSM8123440 NTvKO Control - VTP50469 500nM H3K79me2
GSM8123441 Timecourse Control - DMSO H2AK119ub
GSM8123442 Timecourse Control - VTP50469 500nM 24h H2AK119ub
GSM8123443 Timecourse Control - VTP50469 500nM 8h H2AK119ub
GSM8123444 Timecourse Control - DMSO H3K27me3
GSM8123445 Timecourse Control - VTP50469 500nM 24h H3K27me3
GSM8123446 Timecourse Control - VTP50469 500nM 8h H3K27me3
GSM8123447 Timecourse Control - VTP50469 500nM 16h H3K79me2
GSM8123448 Timecourse Control - VTP50469 500nM 24h H3K79me2
GSM8123449 Timecourse Control - VTP50469 500nM 4h H3K79me2
GSM8123450 Timecourse Control - VTP50469 500nM 8h H3K79me2
GSM8123451 Timecourse Control - DMSO input
GSM8123452 Timecourse Control - DMSO H3K79me2
GSM8549820 MLLAF9 Control - DMSO H3K27me3 rep2
GSM8549821 MLLAF9 Control - VTP50469 500nM H3K27me3 rep2
GSM8549822 MLLAF9 Control - SGC0946 5uM H3K27me3 rep2
GSM8549823 MLLAF9 Control - input

GSM8549824 MLLAF9 Control - DMSO RING1B rep2
GSM8549825 MLLAF9 Control - VTP50469 500nM RING1B
GSM8549826 MLLAF9 Control - SGC0946 5uM RING1B
GSM8549827 MLLAF9 Control - DMSO BCOR
GSM8549828 MLLAF9 Control - VTP50469 500nM BCOR
GSM8549829 MLLAF9 Control - SGC0946 5uM BCOR
GSM8549830 MLLAF9 Control - DMSO H2AK119ub rep3
GSM8549831 MLLAF9 Control - VTP50469 500nM H2AK119ub rep3
GSM8549832 MLLAF9 Control - SGC0946 5uM H2AK119ub rep3
GSM8549833 mESC DRB - DMSO H3K27me3
GSM8549834 mESC DRB - DMSO H2AK119ub
GSM8549835 mESC DRB - DMSO POL2
GSM8549836 mESC DRB - DMSO SUZ12
GSM8549837 mESC DRB - DRB H3K27me3
GSM8549838 mESC DRB - DRB H2AK119ub
GSM8549839 mESC DRB - DRB POL2
GSM8549840 mESC DRB - DRB SUZ12
GSM8549841 mESC DRB - input
GSM8549842 MOLM13 NT - DMSO H3K27me3
GSM8549843 MOLM13 NT - VTP50469 500 nM H3K27me3
GSM8549844 MOLM13 NT - SGC0946 5uM H3K27me3
GSM8549845 MOLM13 PB - DMSO H3K27me3
GSM8549846 MOLM13 PB - VTP50469 500 nM H3K27me3
GSM8549847 MOLM13 PB - SGC0946 5uM H3K27me3
GSM8549848 MOLM13 NT - DMSO H2AK119ub
GSM8549849 MOLM13 NT - VTP50469 500 nM H2AK119ub
GSM8549850 MOLM13 NT - SGC0946 5uM H2AK119ub
GSM8549851 MOLM13 PB - DMSO H2AK119ub
GSM8549852 MOLM13 PB - VTP50469 500 nM H2AK119ub
GSM8549853 MOLM13 PB - SGC0946 5uM H2AK119ub
GSM8549854 MOLM13 NT - input
GSM8549855 MOLM13 NT - DMSO H3K79me2
GSM8549856 MOLM13 NT - SGC0946 5uM H3K79me2
GSM8549857 MOLM13 NT - VTP50469 500 nM H3K79me2
GSM8549858 MOLM13 PB - input
GSM8549859 MOLM13 PB - DMSO H3K79me2
GSM8549860 MOLM13 PB - SGC0946 5uM H3K79me2
GSM8549861 MOLM13 PB - VTP50469 500 nM H3K79me2
GSM8549862 K562 DRB - DMSO H2AK119ub
GSM8549863 K562 DRB - DRB H2AK119ub
GSM8549864 K562 DRB - DMSO H3K27me3
GSM8549865 K562 DRB - DRB H3K27me3
GSM8549866 K562 DRB - input

Genome browser session
(e.g. UCSC)

Provide a link to an anonymized genome browser session for "Initial submission" and "Revised version" documents only, to enable peer review. Write "no longer applicable" for "Final submission" documents.

Methodology

Replicates

Replicates are indicated in the figure legend, in some instances, only one ChIP-seq replicate was performed.

Sequencing depth

All ChIP-seq samples were sequenced to a depth of at least 10million single end reads on a NEXT-seq or NOVA-seq.

Antibodies

DYKDDDDK Tag (D6W5B) Rabbit mAb (New England Biolabs 14793S)
Rabbit anti-MLL1 Antibody (Bio-Strategy BETHA300-086A-M)
Anti-trimethyl-Histone H3 (Lys4) (Merck 07-473)
Rabbit polyclonal to Histone H3 (di methyl K79) - ChIP Grade (ABCAM ab3594-100UG)
Rabbit polyclonal to Histone H3 (acetyl K9) (ABCAM ab4441-100UG)
Rabbit polyclonal to Histone H3 (acetyl K27) (ABCAM ab4729-100UG)
Ubiquityl-Histone H2B (Lys120) (D11) XP® Rabbit mAb (New England Biolabs 5546S)
RING1B (D22F2) XP® Rabbit mAb (New England Biolabs 5694S)
ChIPAb+™ JHDM1B Antibody (Merck 17-10264)
Ubiquityl-Histone H2A (Lys119) (D27C4) XP® Rabbit mAb (New England Biolabs 8240S)
Tri-Methyl-Histone H3 (Lys27) (C36B11) Rabbit mAb (New England Biolabs 9733S)

Peak calling parameters

Broad peaks were called using MACS2 with the following parameters: --SPMR -B -f BAM -g mm --broad. The input non targeting sample bam file was provided as the control (-c).

Data quality

Quality control was performed on the raw sequencing data using FastQC, and the quality scores were found to be within acceptable ranges.

Software

Reads were mapped using Bowtie2, BAM files were converted to TDFs using igvtools ver. 2.4.19 and bigwigs (BamCoverage), then heatmap and profile plots were made using deepTools ver. 3.1.3. Peaks were called using MACS2 ver. 2.1.1.2016309.

Flow Cytometry

Plots

Confirm that:

☒ The axis labels state the marker and fluorochrome used (e.g. CD4-FITC).

☒ The axis scales are clearly visible. Include numbers along axes only for bottom left plot of group (a 'group' is an analysis of identical markers).

☒ All plots are contour plots with outliers or pseudocolor plots.

☒ A numerical value for number of cells or percentage (with statistics) is provided.

Methodology

Sample preparation	Cell lines were collected and cell pellets were resuspended in 100 µL of FACS buffer (cell lines)
Instrument	Flow cytometry was performed using a BD LSRII or LSRFortessa (BD Biosciences), or a Cytek Northern Lights and cell sorting was performed using a BD Fusion3/5 (BD Biosciences)
Software	Analysis was performed using Flowlogic software (Inivai Technologies, Australia) and FlowJo (BD).
Cell population abundance	Sorts for mCherry, BFP and GFP were sorted to near 100% purity prior to experiments.
Gating strategy	Gating was done based on morphology (FSC, SSC) and then for the annexin experiments (Fig. 1i) a representative gating strategy was provided.

☒ Tick this box to confirm that a figure exemplifying the gating strategy is provided in the Supplementary Information.
