# Supplementary material for: DOT1L provides transcriptional memory through PRC1.1 antagonism
Source: Nat Cell Biol. 2026 Feb 3;28(2):307–22. doi: 10.1038/s41556-025-01859-8 (PMC12904788; doi:10.1038/s41556-025-01859-8)

Figure 7n Triplicate blots for PRC1.1

Raw blots in triplicate, only replicate 1 is shown in Figure 7n

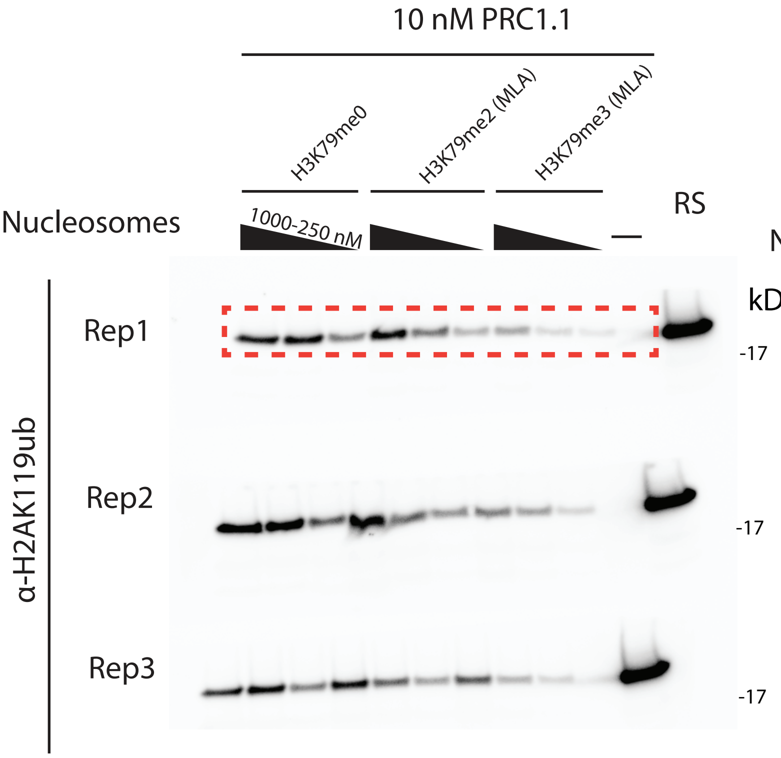

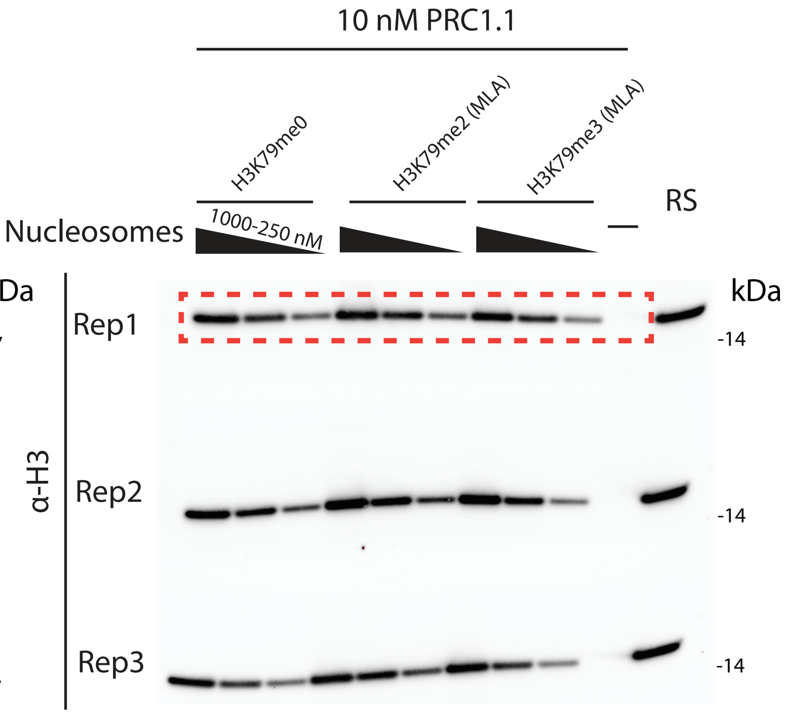

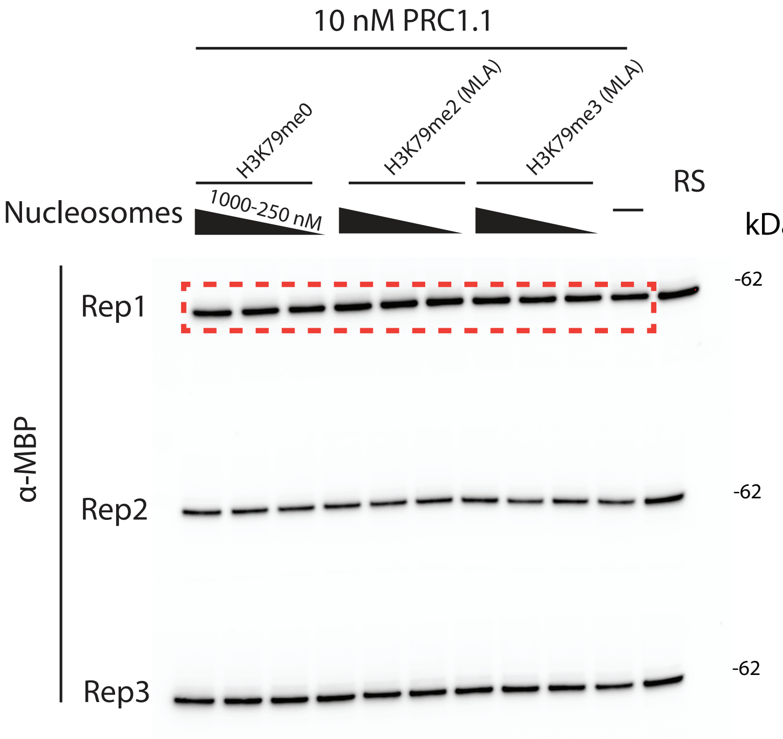

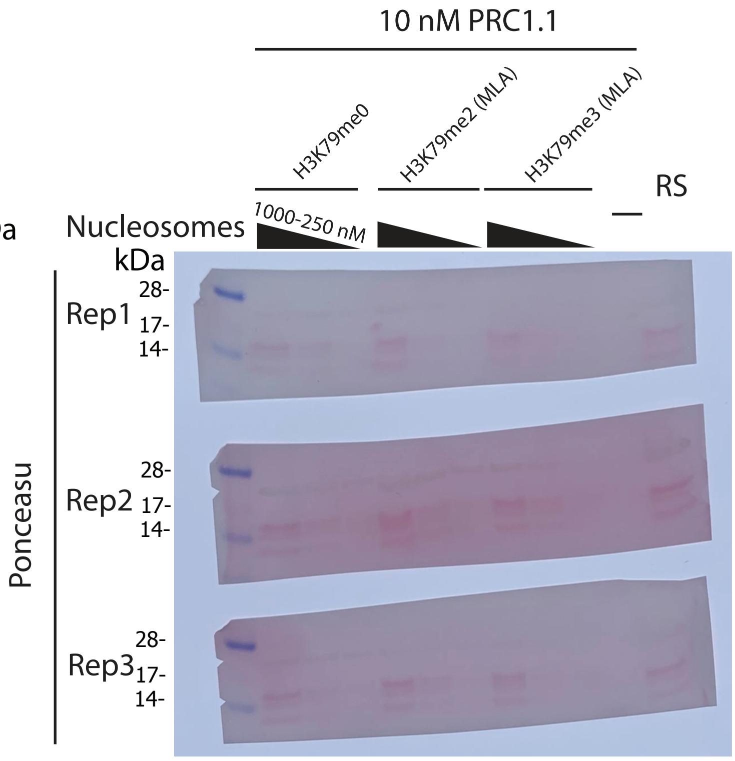

Figure 7n Triplicate blots for PRC1.4

Raw blots in triplicate, only replicate 1 is shown in Figure 7n

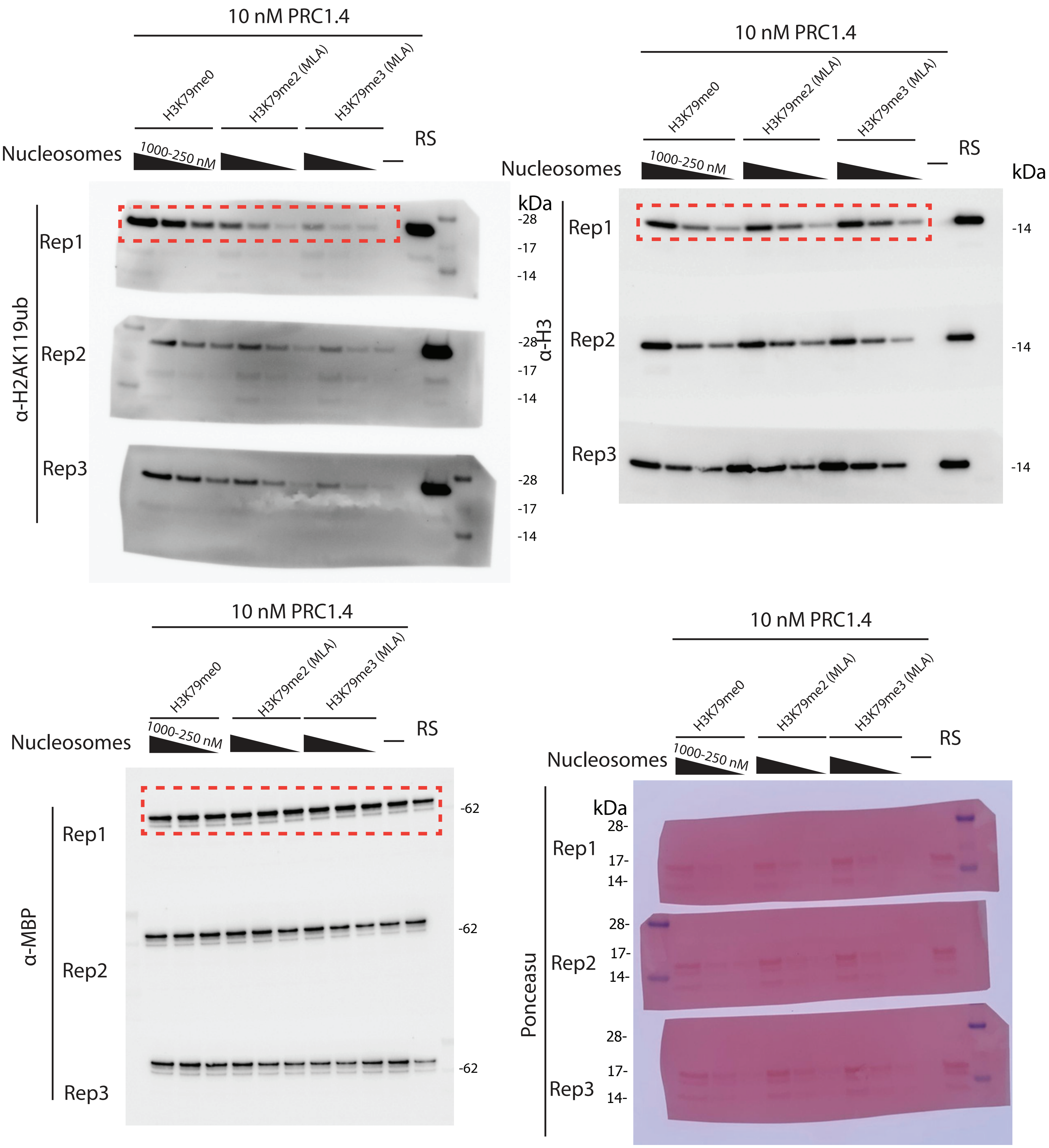

Ext. Data Fig. 1m

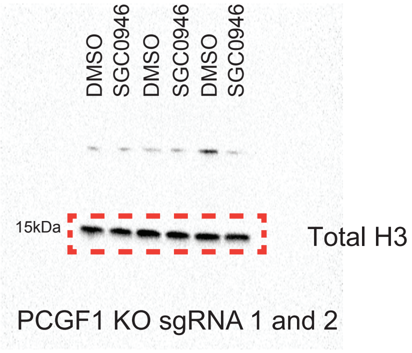

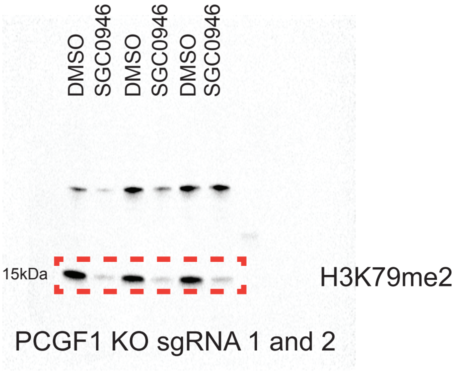

Ext. Data Fig. 8a

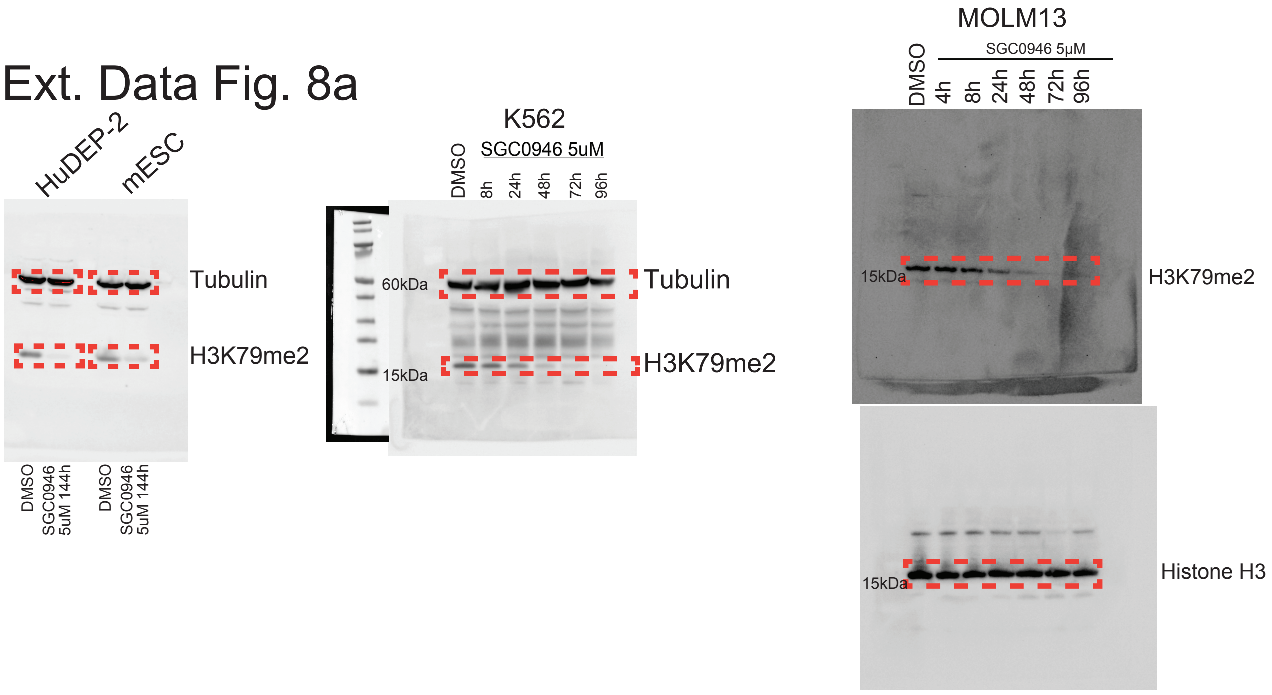

Ext. Data Fig. 9b

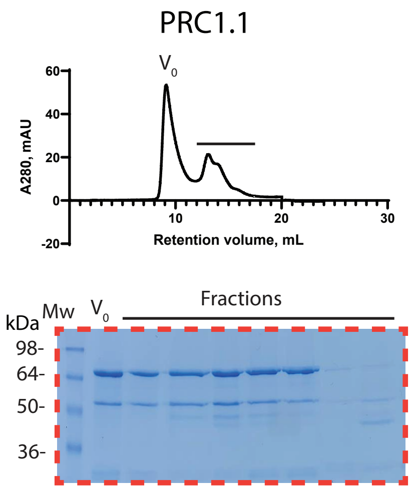

Ext. Data Fig. 9c

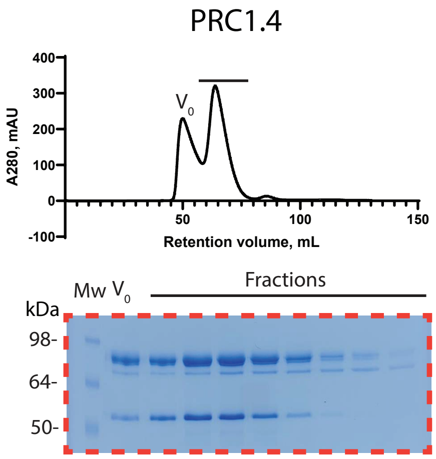

Ext. Data Fig. 9d

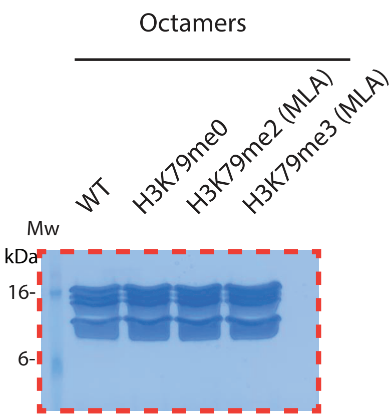

Ext. Data Fig. 9e

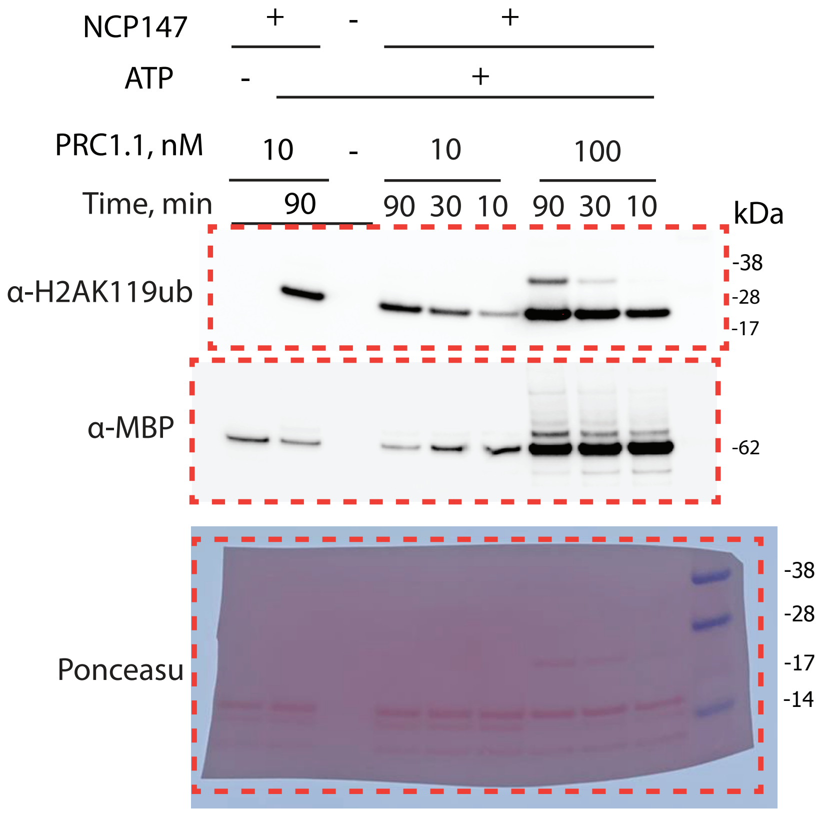

Ext. Data Fig. 9f

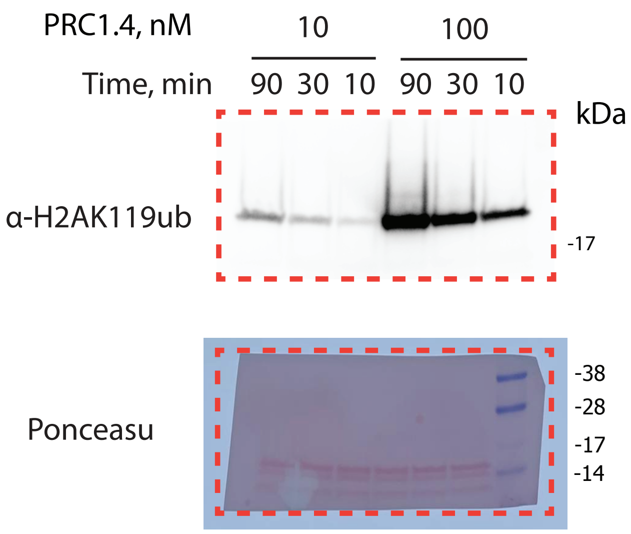

Supplement: Supplementary file 8 — Unprocessed western blots. [file 41556_2025_1859_MOESM8_ESM.pdf]
